# Balance Rehabilitation Unit (BRU™) posturography in benign paroxysmal positional vertigo

**DOI:** 10.1590/S1808-86942012000300017

**Published:** 2015-10-14

**Authors:** Silvia Roberta Gesteira Monteiro, Maurício Malavasi Ganança, Fernando Freitas Ganança, Cristina Freitas Ganança, Heloisa Helena Caovilla

**Affiliations:** a MSc in Sciences - Graduate Program in Human Communication Disorders - Federal University of São Paulo - Paulista School of Medicine (UNIFESP-EPM). Speech and hearing therapist.; b PhD in Otorhinolaryngology - UNIFESP-EPM; Full Professor of Otorhinolaryngology - Federal University of São Paulo - Paulista School of Medicine.; c PhD in Medicine (Otorhinolaryngology) - UNIFESP-EPM; Adjunct Professor of Otology and Neurotology - UNIFESP-EPM.; d PhD - in Sciences - Graduate Program in Human Communication Disorders - Federal University of São Paulo - Paulista School of Medicine (UNIFESP-EPM).; e PhD in Human Communication Disorders - Federal University of São Paulo - Paulista School of Medicine (UNIFESP-EPM). Senior Associate Professor of Otology and Neurotology - UNIFESP-EPM. Otology and Neurotology Program – Federal University of São Paulo – Paulista School of Medicine (UNIFESP-EPM). São Paulo - SP, Brazil.

**Keywords:** ear, inner, postural balance, vertigo, vestibular function tests

## Abstract

Posturography has been used in the evaluation of patients with vestibular disorders.

**Aim:**

To evaluate balance control with the Balance Rehabilitation Unit (BRU™) posturography in patients with Benign Paroxysmal Positional Vertigo. Study design: Prospective case-control.

**Materials and Methods:**

A cross-sectional controlled study was carried out in 45 patients with BPPV, and a homogeneous control group consisting of 45 healthy individuals. Patients were submitted to a balance function evaluation by means of the Balance Rehabilitation Unit (BRU™) posturography.

**Results:**

The mean values of the ellipse area and the sway velocity in a firm surface and saccadic stimulation (*p* = 0.060).

**Conclusion:**

The Balance Rehabilitation Unit (BRU™) posturography enables to identify postural control abnormalities in patients with BPPV.

## INTRODUCTION

Benign Paroxysmal Positional Vertigo (BPPV) is characterized by sudden and quick episodes of vertigo, nausea and/or positional nystagmus upon changes in head position, because of undue presence of calcium carbonate particles, resulting from the fractioning of statoconia from the utricular macula^1^. Head movement causes the shifting of the calcium carbonate particles, causing endolymph acceleration, with consequent abnormal cupule deflexion^2^.

Although BPPV patients primarily have short-duration vertigo fits, postural instability and loss of body balance may also happen in-between fits^3^, ^4^, or after particle repositioning maneuvers^5^. In those cases of persistent vertigo, the disorder may become incapacitating and impair daily life activities; life-quality loss is greater during the fits; although it may also happen outside of the fits; physical aspects are the most altered, followed by the functional and emotional ones^6^.

BPPV is the most common cause of vertigo among adults, representing about 20% of causes of dizziness^7^. The posterior semicircular canal is the most frequently involved (85%-95% of the cases), while the lateral semicircular canal is involved in 5% to 10% of the cases^8^. The diagnosis of BPPV is based on clinical history and it is confirmed by means of nystagmus and vertigo upon positional and positioning maneuvers. Positional nystagmus characteristics upon the Dix–Hallpike^9^ maneuver or positional nystagmus study^10^ point to the involved labyrinth and semicircular canal.

In BPPV, both the oculovestibular reflex - which controls eye movements and gaze stabilization, and the vestibulospinal reflex - which keeps body balance stable, are involved^4^. Nonetheless, most of the times, since the specialist's attention is focused on the vertigo, the body instability, ataxia and a tendency to fall complaints are neglected^11^. These complaints establish that BPPV patients should be submitted to a more thorough neurotological evaluation^5^.

As part of such assessment, posturography supplies information, not only from the vestibular system, but also from the multisensory system, which contribute to maintain body balance, and it may provide information which is not detected upon electronystagmography^12^. Static posturography assesses the vestibulospinal reflex, analyzing body sway with the patient standing up within the confines of the center of gravity, and it helps study the balance of patients with positional dizziness^13^. The posturography module of the Balance Rehabilitation Unit (BRU™), which utilizes visual stimuli projected onto virtual reality goggles, provides information about the position of the patient's pressure center in ten sensorial situations by means of measuring the areas of stability threshold, the shifting area of the pressure center (ellipse area) and sway velocity^14^.

We carried out the present study because we did not find references concerning posturography in Balance Rehabilitation Unit (BRU™), upon assessing body balance in BPPV patients in comparison with the control group made up of healthy individuals. Moreover, the possible implications of our findings in the rehabilitation of patients with this disorder were previously shown by the identification of an increase in stability and reduction in the elliptical area on firm surface with the eyes closed, upwards and downwards optokinetic bars and horizontal visual-vestibular interaction after the Epley's maneuver in elderly with BPPV^15^.

The goal of the present study is to assess body balance upon Balance Rehabilitation Unit (BRU™) posturography in patients with benign paroxysmal positional vertigo.

## MATERIALS AND METHODS

This study was previously approved by the Ethics in Research Committee of our institution (Protocol # 2010/07). All the patients signed an informed consent form prior to the beginning of the study.

In this controlled cross-sectional study, the sample was made up of an experimental group of 45 adult male or female patients diagnosed with BPPV and a homogeneous control group, made up of 45 healthy individuals.

Patient inclusion criteria in the experimental group were: a diagnosis of benign paroxysmal postural vertigo, established by the ENT physician, according to the Dix-Hallpike (1952) test and/or the positional nystagmus test in right and left-side lateral decubitus and not having been submitted to a prior treatment in the past six months.

We took off the study those patients with BPPV incapable of understanding and following a simple verbal command; who could not independently stay up straight; with severe vision involvement or vision impaired without corrective lenses; with orthopedic disorders which limit movements; with lower limb prostheses; with neurological and/or psychiatric diseases; those who ingested alcohol 24 hours before the test; using substances which act on the central nervous system or the vestibular system; and those patients submitted to body balance rehabilitation exercises in the past semester.

The experimental group patients were submitted to a neurotological assessment to characterize the patient's neurological status. This assessment was made up by anamneses; visual inspection of the external acoustic meatus; tonal audiometry; speech recognition threshold; speech recognition percentage index; tympanometry; acoustic reflex study; Brazilian version of the Dizziness Handicap Inventory^16^, ^17^; dizziness analog scale^18^; positioning and positional nystagmus test; ocular movement calibration; spontaneous and semi-spontaneous nystagmus; fixed and randomized saccadic movements; pendulum tracking; optokinetic nystagmus; decreasing rotational pendulum test, caloric test; and Balance Rehabilitation Unit (BRU™)^19^ posturography.

The control group patients were submitted to interview, in order to characterize the lack of neurotological symptoms, and Balance Rehabilitation Unit (BRU™) posturography.

The Balance Rehabilitation Unit (BRU™) is made up of a computer and a software; a metal structure; support with loops and protection belt; force platform; virtual-reality goggles; accelerometer and foam pillow^14^.

The Balance Rehabilitation Unit (BRU™)^19^ posturography was carried out with the patient standing up on the pad, barefoot and with the arms resting along the body. The left and right internal malleoli were placed on the ends of the intermalleolus line and the two first fingers remained spread 10º from the midline. Spectacles use was allowed, when needed. The midpoint of the intermalleolus line was established as the limit center of the stability circle pattern. The posturography mode provided information about the position of the patient's pressure center by means of the measures of the stability limit area, the ellipse area and sway velocity.

The stability threshold was established by asking the patient to shift his body anteroposteriorly and laterally, employing the ankle strategy, without moving his feet or using trunk strategies. The 95% confidence ellipse area and the sway velocity were measured with the patient standing still for 60 seconds, standing up on the pad in ten sensorial situations: 1) open eyes; 2) closed eyes; 3) on medium density foam pillow and closed eyes; 4) saccadic stimulation; 5) left-to-right horizontal optokinetic stimulation; 6) right-to-left horizontal optokinetic stimulation; 7) top-to-bottom vertical optokinetic stimulation; 8) bottom-to-top vertical optokinetic stimulation; 9) horizontal optokinetic stimulation associated to slow and uniform rotational head movements; 10) vertical optokinetic stimulation associated to slow and uniform flexion and extension of the head. The virtual reality goggles were worn as of the fourth situation.

We carried out a descriptive statistical analysis in order to characterize the sample. The Fisher's exact test was used in order to test gender ratio homogeneity between the groups. The T-Student test was used in the comparative analysis between the groups for age and stability limit, because of symmetry and adherence to the normal distribution to the normality Kolmogorov-Smirnov test. The T-Student test was used to check for differences between the sway velocity mean values and the ellipse area in the Balance Rehabilitation Unit (BRU™) situation between the BPPV experimental group and the control group, because the normality assumption was rejected by the Kolmogorov-Smirnov test, and these variables were transformed by means of the logarithmical function. In those situations showing a significant difference between the groups, we calculated the test's power. The values found vary between 63% and 100%, proving that the sample size was enough in order to attain tests with 80% power.

The analyses were carried out by the SPSS 10.0 for Windows (Statistical Package for Social Sciences, version 10.0, 1999) software; the significance level adopted for the statistical tests was 5%(α =0.05)

## RESULTS

We assessed 45 individuals in the control group and 45 patients with a diagnostic hypothesis of BPPV, with vertigo and positional nystagmus upon the Dix-Hallpike diagnostic test. The control group was made up of nine male individuals (20.0%) and 36 (80.0%) females. The group of BPPV patients was made up by 12 male individuals (26.7%) and 33 (73.3%) females. We did not find statistically significant differences between the groups as far as gender is concerned (*p* = 0.619).

As to age, the control group had mean age of 45.62 ± 11.84 years, and the experimental group had mean age of 49.13 ± 9.53 years. There were no statistically significant differences between the groups as far as age is concerned (*p* = 0.121).

The patients with BPPV were classified according to the semicircular canal involved, based on the positional nystagmus characteristics found upon the Dix-Hallpike maneuver. The experimental group was made up of 18 patients (40.0%) with involvement of the right posterior semicircular canal; 17 (37.8%) with involvement of the left posterior semicircular canal; five (11.1%) with involvement of the right and left posterior semicircular canal; two (4.4%) with involvement of the left anterior semicircular canal; one (2.2%) with involvement of the right anterior semicircular canal; one (2.2%) with involvement of the right lateral semicircular canal and one (2.2%) with involvement of the right posterior and lateral posterior semicircular canals. The 45 patients from the experimental group had the physiopathology of canalithiasis.

Dizziness characterized as to periodicity, triggering position and postural instability is depicted on Table 1. In 14 patients (31.1%) dizziness started over 5 years in the past; in 13 (28.9%), from seven months to one year; in 11 (24.4%), in up to six months; in five (11.1%), from three to four years; and, in two (4.4%), from 13 months to two years.

**Table 1:** Characterization of BPPV patients as to periodicity, triggering position and body instability complaint.

Categories		Absolute Frequency (N)	Relative Frequency (%)
Periodicity	Daily	31	68.9
	Weekly	8	8.0
	Monthly	6	6.0

Triggering position	Laying down	30	66.7
	Standing up	25	55.6
	Right-side decubitus	27	60.0
	Left-side decubitus	23	51.1
	Head hyperextension	18	40.0
	Lean forward	16	35.6

Postural instability	Presence	38	84.0
	Absence	7	15.6

The score mean value upon the use of the dizziness analog scale was 7.24 points (standard deviation = 1.51), the minimum was four and the maximum was 10.

The mean score upon the use of the quality of life questionnaire was 49.11 points (standard deviation = 21.37) for the total score, 17.82 points (standard deviation = 6.36) for the physical aspect, 12.98 points (standard deviation = 9.82) for the emotional aspect and 18.40 points (standard deviation = 9.18) for the functional aspect.

Upon vestibulometry, 24 patients (53.3%) had results within normal ranges and 21 (46.6%) had peripheral changes, of whom seven had vestibular hypofunction in the caloric test.

Upon the Balance Rehabilitation Unit (BRU™) posturography, there were no statistically significant differences (*p* = 0.597) between the values of the stability limit area (cm^2^) for the control group (mean = 183.24; standard deviation = 49.94; median = 190.00; variation = 77-277) and those from the BPPV group (mean = 189.53; standard deviation = 61.92; median = 179.00; variation = 35-338).

Table 1 depicts the characterization of BPPV patients as to periodicity, triggering position and body instability complaint.

Table 2 depicts descriptive values and the comparative analysis concerning sway velocity (cm/s) and ellipse area (cm^2^) in the control group and that with BPPV patients. The mean sway velocity values of the experimental group were greater than those in the control group in all situations assessed; with statistically significant differences (*p* < 0.05), except for the firm surface and saccadic stimulation (*p* = 0.060) situations. The mean values from the ellipse area in the experimental group were higher than those from the control group in all situations assessed, with significant differences (*p* < 0.05).

**Table 2:** Mean values, standard deviation and sway velocity p-values and ellipse areas in the ten situations of patients with BPPV and the control group.

BRU™ Sensorial situations	Groups	Sway velocity cm/s			Ellipse area cm^2^		
		Mean	Standard deviation	*p*-value	Mean	Standard deviation	*p*-value
1. FS/OE/without stimulus	BPPV	1.05	0.76	< 0.001*	5.54	7.04	< 0.001*
	control	0.73	0.28		1.64	0.97	
2. FS/CE	BPPV	1.47	1.03	< 0.001*	6.86	8.764	< 0.001*
	Control	0.91	0.29		1.78	1.15	
3. Foam/CE	BPPV	3.40	2.32	0.012*	17.062	17.49	< 0.001*
	Control	2.51	0.93		7.59	4.18	
4. FS/Saccadic	BPPV	1.19	0.57	0.06	4.06	4.78	< 0.001*
	Control	0.99	0.42		1.43	1.10	
5. FS/Bars/right-side opto	BPPV	1.23	0.85	0.015*	5.65	9.46	< 0.001*
	Control	0.90	0.27		1.55	1.00	
6. FS/left-side opto bar	BPPV	1.26	0.68	0.002*	6.3	7.74	< 0.001*
	Control	0.91	0.30		1.49	1.03	
7. FS/Downwards opto bar	BPPV	1.13	0.52	0.025*	4.41	5.34	< 0.001*
	Control	0.91	0.30		1.56	1.42	
8. FS/Upwards opto bar	BPPV	1.25	0.70	0.009*	5.00	6.14	<0.001*
	Control	0.93	0.32		1.78	1.56	
9. FS/Visual-vestibular/horizontal interaction	BPPV	1.60	0.65	<0.001*	7.55	10.21	< 0.001*
	Control	1.17	0.43		2.22	1.74	
10. FS/Visual - Vestibular/Vertical interaction	BPPV	1.58	0.55	0.004*	4.81	4.12	< 0.001*
	Control	1.28	0.41		2.32	1.52	

BRU™ (Balance Rehabilitation Unit) / Firm Surface (FS) / Open Eyes (OE) / Closed Eyes (CE) / Benign Positional Paroxysmal Vertigo (BPPV). Student T test Level of significance < = 0.05.

* significant test.

## DISCUSSION

Recent developments in understanding and treating benign paroxysmal positional vertigo are welcome, since this is one of the most prevalent conditions in patients looking for neurotology care.

In the assessment of 45 BPPV patients, before treatment maneuvers, there was a prevalence (88.9% of the cases) of posterior semicircular canal involvement. Similarly to our findings, the literature also reports a prevalence of posterior semicircular canal involvement in BPPV patients^8^, ^20^, ^21^, ^22^.

The postural instability complaint was present in most of the cases (84.0%); in agreement with the statement that vestibular disorder symptoms in BPPV patients include not only the vertigo and unbalance fits caused by a sudden change in head position, but there is also an increase in postural sway during the vertigo episodes^4^.

The Brazilian version^17^ of the Dizziness Handicap Inventory^16^ used indicated a moderate influence (mean of 49.11 points) of symptoms in quality of life^23^, similarly to what was previously found (mean of 52.89 points) in BPPV patients before treatment^6^.

The mean value of 7.24 points upon the dizziness analog scale^18^ suggested severe dizziness in the series evaluated. We did not find references concerning the use of the BPPV patient analog scale.

Peripheral changes (46,6%) were prevalent in the vestibular function test, including the vestibular hyporeflexia of the same labyrinth affected by the BPPV in five patients and of both labyrinths in two. Vestibular hypofunction was reported by other authors in BPPV patients^24^, ^25^, ^26^, ^27^.

The Balance Rehabilitation Unit (BRU™) posturography showed that the stability limit area values were similar to those in the control group, indicating that our patients with BPPV had stability to move their body mass center and keep balance without changing the support basis.

The ellipse area and the sway velocity mean values in the experimental group were higher than those in the control group in all situations assessed, except for the sway velocity on the firm surface and stimulation with saccadic movements. Therefore, patients with BPPV were unable to keep postural balance with and without vision deprivation or visual conflict; proprioceptive and vestibular-visual interaction. Similarly to our findings, patients with BPPV submitted to other types of static or dinamic posturogram^4^, ^5^, ^13^, ^28^, ^29^, ^30^, ^31^, ^32^ also had postural stability involvement with and without vision and upon inaccurate somatosensory information. On the other hand, in BPPV, when the static posturography is normal, one may assume compensation by a replacement mechanism, in which the patient uses all the possible sensorial information; and, when the values are found altered, there is visual and proprioceptive deprivation or disturbance^33^.

The Balance Rehabilitation Unit (BRU™) posturography proved to be a method able to identify changes in the sensory system associated with body balance in BPPV patients. Findings concerning sway velocity value changes and elliptical areas may be considered of great usefulness in the battery of tests which make up the neurotological assessment.

## CONCLUSION

The Balance Rehabilitation Unit (BRU™) posturography enables the identification of abnormalities in the sway velocity values and ellipse area with and without vision, visual conflict, somatosensory conflicts and visual-vestibular interaction in patients with BPPV.
